# Assessment of kallikrein 6 as a cross-sectional and longitudinal biomarker for Alzheimer’s disease

**DOI:** 10.1186/s13195-018-0336-4

**Published:** 2018-01-29

**Authors:** Kalicharan Patra, Antoninus Soosaipillai, Sigrid Botne Sando, Camilla Lauridsen, Guro Berge, Ina Møller, Gøril Rolfseng Grøntvedt, Geir Bråthen, Ilijana Begcevic, Simon Moussaud, Lennart Minthon, Oskar Hansson, Eleftherios P. Diamandis, Linda R. White, Henrietta M. Nielsen

**Affiliations:** 1 Department of Neurochemistry, Stockholm University, Svante Arrhenius väg 16B, 106 91 Stockholm, Sweden; 2 Department of Pathology and Laboratory Medicine, Lunenfeld-Tanenbaum Research Institute-Mount Sinai Hospital, Toronto, ON Canada; 3 Department of Neurology, University Hospital of Trondheim, Trondheim, Norway; 4 Department of Neuromedicine and Movement Science, Norwegian University of Science and Technology, Trondheim, Norway; 5 Department of Clinical Sciences, Lund University, Lund, Sweden

**Keywords:** KLK6, Neurosin, Biomarker, Neurodegeneration, Alzheimer’s disease

## Abstract

**Background:**

Kallikrein 6 (KLK6) is known to be an age-related protease expressed at high levels in the central nervous system. It was previously shown to be involved in proteolysis of extracellular proteins implicated in neurodegenerative diseases such as Alzheimer’s disease (AD), prompting validation of KLK6 as a potential biomarker of disease. However, analyses of both plasma and cerebrospinal fluid (CSF) levels of KLK6 in patients with AD have been inconclusive. We present a detailed analysis of KLK6 in plasma and CSF in two separate cohorts in a cross-sectional and a longitudinal clinical setting.

**Methods:**

The cross-sectional cohort included control subjects without dementia and patients with AD, and the longitudinal cohort included patients with MCI and patients with AD followed over a 2-year period. Plasma and CSF levels of KLK6 were quantified by use of a previously developed and validated enzyme-linked immunosorbent assay. Statistical analyses were performed to compare KLK6 levels between diagnostic groups and to identify potential associations between KLK6 level, age, apolipoprotein E (*APOE*) genotype, total apoE level and the classical CSF AD biomarkers.

**Results:**

In the cross-sectional setting, KLK6 levels in plasma but not in CSF were significantly higher in the AD group than in control subjects. CSF but not plasma KLK6 levels were positively correlated with age in both the cross-sectional and longitudinal settings. In both cohorts, the CSF KLK6 levels were significantly and positively correlated with the CSF levels of core AD biomarkers. Total plasma and CSF apoE levels were positively associated with KLK6 in the cross-sectional study. Finally, during the 2-year monitoring period of the longitudinal cohort, CSF KLK6 levels increased with disease progression over time in the investigated patient groups.

**Conclusions:**

In two separate cohorts we have confirmed the previously reported correlation between age and CSF levels of KLK6. Increased plasma KLK6 levels in patients with AD with a more advanced disease stage suggest KLK6 as a potential biomarker in patients with AD with more severe dementia. Significant correlations between KLK6 levels and core CSF AD biomarkers suggest molecular links between KLK6 and AD-related pathological processes.

## Background

Alzheimer’s disease (AD) is the most common form of age-related dementia. It affects over 46 million individuals worldwide, and the incidence is estimated to double every 20 years according to a 2017 Alzheimer’s Association report [1]. Despite the improved diagnostic tools available to clinicians, including cerebrospinal fluid (CSF) biomarkers [2], a definite diagnosis of AD can still only be made on the basis of a neuropathological assessment [3]. Importantly, more efficient biomarkers, preferably in plasma owing to the less invasive sampling procedure, are needed to assess the risk of developing AD and to identify patients who will convert from mild cognitive impairment (MCI) to AD. Ultimately, biomarkers per se should offer a window to the pathological processes coupled to the development and progression of AD [4] and clearly distinguish between these processes and those related to normal aging. Aging alone is one of the major risk factors for AD [5], but little is known about the molecular processes that distinguish healthy aging from pathological aging [6]. In addition to accumulation of amyloid-β (Aβ) peptides and hyperphosphorylated tau, other age-related alterations such as processes linked to inflammation are believed to play an important role in AD pathogenesis [7, 8]. Furthermore, the presence of the apolipoprotein E (*APOE*) ε4 allele, the strongest genetic risk factor for sporadic AD [9], may affect the course of aging in individuals carrying this AD risk allele [10]. Owing to the heterogeneity in the age at onset of sporadic AD, future efforts need to address whether a different or additional set of biomarkers would be more appropriate for patients with AD developing disease at a younger or older age.

Kallikrein 6 (KLK6) is a protein highly expressed in the brain and spinal cord, with levels in body fluids increasing with age [11–13]. This age association was proposed to be disrupted in AD [14]. These initial findings led to the assessment of KLK6 as a candidate biomarker for AD for which plasma, CSF and tissue levels of human KLK6 were analysed. Diamandis and colleagues pioneered the field of KLK6 quantification and developed an immunofluorometric assay to assess human KLK6 levels [15–17]. Menendez-Gonzalez and co-authors reported a decrease in plasma KLK6 in subjects with AD compared with age-matched control subjects [13] but no significant alteration between patients with AD and patients with MCI [18]. Researchers in another study reported significantly decreased CSF KLK6 levels in patients with synucleinopathies, but not in patients with AD [19]. Hence, consensus remains to be reached on the potential usefulness of KLK6 fluid levels as a biomarker for AD.

In this study we analysed KLK6 levels in plasma and CSF from subjects included in two separate cohorts including patients with AD and control subjects using a previously developed and validated quantification method. We hypothesised that the discrepancy in reported KLK6 levels results from the use of different and not well-standardised quantification methods, as well as from possible age differences in previously examined cohorts. To address these issues, we used a previously developed and validated quantification method to quantify CSF and plasma levels of KLK6 in a cross-sectional study of older patients with AD with a more advanced disease stage and a longitudinally followed cohort including patients with amnestic MCI and patients with sporadic AD with disease onset before and after 65 years of age. In addition, we analysed potential associations between KLK6 levels; age; *APOE* genotype; total apoE level (assessed in the cross-sectional setting only); and the AD biomarkers amyloid-β 1–42 (Aβ_42_), amyloid-β 1–40 (Aβ_40_), total tau (t-tau) and phosphorylated tau (p-tau).

## Methods

### Study subjects

A total of 227 subjects were analysed in 2 cohorts. All subjects were grouped on the basis of clinical criteria only, without an influence of CSF AD biomarker profiles. The described studies were performed in accordance with the Helsinki declaration.

Descriptive results of the cross-sectional study (cohort 1) were previously reported [20]. In brief, subjects in cohort 1 were recruited and examined at the Memory Clinic at Skåne University Hospital, Malmö, Sweden, between the years 2001 and 2008. Subjects diagnosed with AD met the criteria of the *Diagnostic and Statistical Manual of Mental Disorders, Third Edition–Revised*, for dementia [21] and the National Institute of Neurological and Communicable Disorders and Stroke-Alzheimer’s Disease and Related Disorders Association (NINCDS-ADRDA) criteria for probable AD [22]. Subjects in the control group sought medical attention for experienced subjective cognitive symptoms but were found not to be affected by dementia or other neurological disease after a detailed clinical investigation. Following lumbar puncture, CSF was collected into polypropylene tubes, centrifuged, aliquoted and frozen at −80 °C within 1 h of collection. Blood was collected into ethylenediaminetetraacetic acid-containing tubes, followed by centrifugation at 2000 × *g* for 10 minutes at 4 °C, and then plasma was aliquoted and frozen at −80 °C pending analysis. *APOE* genotyping and CSF AD biomarker profiling were performed during clinical routine [20]. Informed consent was obtained from all participants, and the study protocol was approved by the local ethics committee at Lund University, Sweden.

Subjects recruited into the second cohort enrolled in a 24-month longitudinal study performed at the Department of Neurology, University Hospital, Trondheim, Norway, between 2009 and 2015. All study subjects were examined by two neurologists according to procedures described elsewhere [23]. Briefly, at inclusion, patients diagnosed with AD met the NINCDS-ADRDA criteria and patients with amnestic MCI met the International Working Group on Mild Cognitive Impairment criteria [24]. Control subjects were genetically unrelated to the patients and were either recruited from societies for retired people or volunteering caregivers of the included patients. All control subjects underwent lumbar puncture only at baseline, due to local ethics restrictions, but they participated in neuropsychological examinations at baseline and after 2 years. Assessment of patients with MCI or AD included reexamination with lumbar puncture and neuropsychological evaluation at 12 and 24 months after inclusion. Accordingly, patients were divided into three groups: patients with MCI who remained unchanged over 24 months (MCI-MCI), patients with MCI who developed AD (MCI-AD) and patients diagnosed with AD at inclusion (AD-AD). Cerebrospinal fluid samples were directly collected into polypropylene cryovials and frozen at −80 °C within 30 minutes without centrifugation. In this cohort, plasma was collected following centrifugation at 1500 × *g* for 10 minutes at room temperature. Erythrocyte counts were (mean ± SD) 1.6 ± 2.8/μL (range 0–17 erythrocytes/μL). *APOE* genotyping [25] and core AD biomarker assays were performed in-house according to the manufacturer’s recommendations [23]. All subjects included in the longitudinal study, or their proxies, gave written informed consent. The biobank is licensed by the Norwegian Directorate for Social and Health Affairs, and the protocol was approved by the Regional Committee for Medical Research Ethics.

### Measurement of KLK6 levels

Plasma and CSF samples were aliquoted and shipped on dry ice to the Advanced Centre for Detection of Cancer Laboratory at Mount Sinai Hospital, Toronto, Ontario, Canada, on two different occasions for cohort 1 and cohort 2. An enzyme-linked immunosorbent assay (ELISA) developed in-house was employed to assess the levels of KLK6 in plasma and CSF samples. It detects pro-KLK6 and active KLK6 with similar potency. For assay details, see the publication by Shaw and Diamandis [26]. Briefly, samples were randomised (to avoid any analytical bias) and analysed in duplicate with a sandwich ELISA using a mouse monoclonal antibody against human KLK6 as a capture antibody and another mouse monoclonal biotinylated antibody against human KLK6 for detection. White polystyrene microtitre wells were coated with 500 ng of capture antibody in 100 μL of coating buffer (50 mmol/L Tris-HCl, pH 7.8) and incubated for 14–16 h; washed in wash buffer (10 mmol/L Tris-HCl buffer, 150 mmol/L NaCl and 0.5 mL/L Tween 20); and incubated for 2 h with samples diluted in 6% bovine serum albumin, 25 mL/L mouse serum, 100 mL/L goat serum and 10 g/L bovine immunoglobulin G. Plates were washed as before, incubated with 50 ng (100 μL) of detection antibody for 1 h, washed, and incubated again with 5 ng (100 μL) of alkaline phosphatase-conjugated streptavidin. Diflunisal phosphate was used as a substrate for phosphatase activity, and fluorescence was measured with a time-resolved fluorometer. Calibration and data reduction were performed as described elsewhere [27]. The inter-assay coefficient of variation was less than 4% for plasma and CSF analyses. Recovery percentages were 101–110% for plasma and 102–110% for CSF.

### Statistical analysis

All statistical analyses were performed using JMP software version 12.1.0 (SAS Institute, Cary, NC, USA). A *p* value < 0.05 was considered statistically significant. Normal distribution of the data was evaluated by use of the Shapiro-Wilk test. Accordingly, statistical tests employed for group comparisons were either non-parametric (Kruskal-Wallis test followed by pairwise comparisons with the Mann-Whitney *U* test for plasma KLK6 in cohort 1) or parametric (analysis of variance [ANOVA] followed by pairwise comparisons with Student’s *t* test for CSF KLK6 in cohort 1 and for plasma and CSF KLK6 in cohort 2). The Bonferroni correction was used to account for multiple comparisons with the number of comparisons indicated where applicable. Similarly, parametric (Pearson’s correlation test) or non-parametric (Spearman’s rho) tests were used to assess potential correlations between plasma and CSF KLK6 levels with age; Mini Mental State Examination (MMSE) score; and CSF levels of CSF t-tau, CSF p-tau, CSF Aβ_42_ and CSF Aβ_40_. In cohort 2 CSF KLK6 levels were positively associated with age, which was accounted for when performing group comparisons by use of analysis of covariance (ANCOVA) for age correction. Multivariate analysis of variance (MANOVA) was used to assess group-specific changes in KLK6 levels over time in cohort 2. In graphs for the latter analysis, data were presented as least squares mean values.

## Results

### Patient demographics and characteristics

The cross-sectional cohort (cohort 1) had a total of 86 participants, grouped as control subjects without dementia (non-AD, *n* = 43; median age, 61 years) and subjects with AD (*n* = 43; median age, 78 years). The AD patient group was significantly different (*p* < 0.001) from the control group in age at examination, MMSE scores and baseline CSF levels of core AD biomarkers such as t-tau, p-tau and Aβ_42_ (Table 1). The cohort specifics were previously reported [20].

**Table 1:** Demographics and clinical characteristics

Characteristics	Control group (*n* = 43)	AD group (*n* = 43)	*p* Value
Sex, M/F, %	47/53	36/64	
Age at examination, years^a^	61 (43–80)	78 (60–94)	<0.001
MMSE score^a^	29 (24–30)	18 (4–23)	< 0.001
*APOE* ε4 status (−/−, +/−, +/+)	33, 8, 2	12, 24, 7	
CSF Aβ_42_, pg/mL^a^	539 (200–990)	335 (140–530)	< 0.001
CSF t-tau, pg/mL^a^	323 (89–690)	700 (360–1990)	< 0.001
CSF p-tau, pg/mL^a^	41 (20–87)	88 (44–226)	< 0.001

*Abbreviations: AD* Alzheimer’s disease, *APOE* Apolipoprotein E, *Aβ*_*42*_ Amyloid-β 1–42, *CSF* Cerebrospinal fluid, *MMSE* Mini Mental State Examination, *p-tau* Phosphorylated tau, *t-tau* Total tau

Age at examination, MMSE scores, Aβ_42_, t-tau and p-tau are presented as median with range

*p* Values reported are derived from non-parametric comparisons of each pair using Wilcoxon’s method. *APOE* ε4 genotype status is presented as null (−/−), heterozygous (+/−) and homozygous (+/+)

^a^Median (minimum–maximum)

The longitudinal cohort (cohort 2) consisted of 141 participants divided into 4 groups on the basis of re-examination after 24 months: control subjects (*n* = 58; median age, 67 years), patients with MCI-MCI (*n* = 27; median age, 64 years), patients with MCI-AD within 24 months (*n* = 28; median age, 64 years) and patients with AD-AD (*n* = 28; median age, 64 years). The four groups differed significantly in MMSE scores (*p* < 0.0001) and average baseline CSF concentrations of Aβ_42_ (*p* < 0.0001), Aβ_40_ (*p* = 0.02), t-tau (*p* < 0.0001) and p-tau (*p* < 0.0001). No significant difference in age was found between the patient groups included in cohort 2, but the MCI-AD and AD-AD groups differed significantly in age compared with control subjects (*p* = 0.0036 for MCI-AD and *p* = 0.0228 for AD-AD, after Bonferroni correction for six comparisons). Although the MCI-MCI group had the same median age as the other two patient groups, the difference in age from control subjects was not significant (*p* = 0.2989) (Table 2).

**Table 2:** Demographic and clinical characteristics at baseline

Characteristics	Control subjects (*n* = 58)	MCI-MCI (*n* = 27)	MCI-AD (*n* = 28)	AD-AD (*n* = 28)	*p* Value
Sex, M/F, %	32/68	56/44	42/58	50/50	
Age at examination, years^a^	67 (57–77)	64 (53–78)	64 (56–71)^b^	64 (54–78)^c^	0.0016
MMSE score^a^	30 (28–30)	28 (25–30)^d^	27 (23–29)^d^	23 (16–27)^d^	< 0.0001
*APOE* ε4 status (−/−, +/−, +/+)	32, 19, 0	7, 3, 7	5, 8, 11	5, 16, 7	
CSF Aβ_42_, pg/mL^a^	970 (499–1674)	647 (173–1065)^d^	494 (283–1060)^d^	460 (212–1092)^d^	< 0.0001
CSF Aβ_40_, pg/mL^a^	17,134 (11,152–40,851)	12,554 (3553–31,343)^c^	15,419 (8021–23,258)	15,397 (6008–29,090)	0.0056
CSF t-tau, pg/mL^a^	269 (137–1314)	280 (98–1057)	533 (163–2325)^d^	669 (222–1540)^d^	< 0.0001
CSF p-tau, pg/mL^a^	53 (33–135)	54 (16–131)	87 (37–169)^d^	93.0 (36–157)^d^	< 0.0001

*Abbreviations: AD* Alzheimer’s disease, *APOE* Apolipoprotein E, *Aβ*_*42*_ Amyloid-β 1–42, *CSF* Cerebrospinal fluid, *MMSE* Mini Mental State Examination, *p-tau* Phosphorylated tau, *t-tau* Total tau, *AD-AD* Patients diagnosed with Alzheimer’s disease at inclusion, *Aβ*_*40*_ Amyloid-β 1–40, *MCI-AD* Patients with mild cognitive impairment who developed Alzheimer’s disease, *MCI-MCI* Patients with mild cognitive impairment that remained unchanged over 24 months

Sex is presented as percentage male vs female; age at inclusion to the study, MMSE scores, t-tau, p-tau, Aβ_42_ and Aβ_40_ levels are presented as median with range. Statistical significance for differences in MMSE scores and levels of CSF biomarkers upon pairwise comparison of control subjects with the MCI-MCI, MCI-AD and AD-AD groups is presented after Bonferroni correction (*n* = 6). *APOE* ε4 genotype status is presented as null (−/−), heterozygous (+/−) and homozygous (+/+). *p* Value is reported for non-parametric analysis of variance among groups by Wilcoxon/Kruskal-Wallis test

^a^Median (minimum–maximum)

^b^*p* < 0.05

^c^*p* < 0.01

^d^*p* < 0.001

### Higher plasma KLK6 levels in patients with AD included in cohort 1

**Fig. 1:**
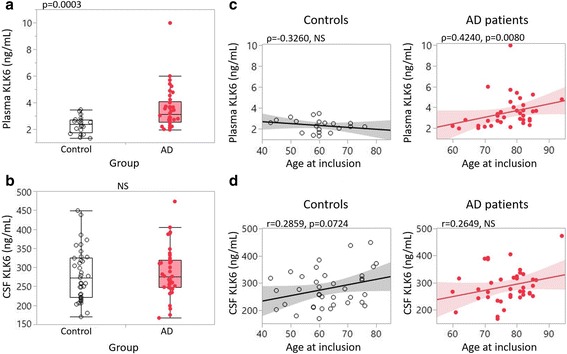
Kallikrein 6 (KLK6) levels between subject groups and association with age in cohort 1. **a**, **b** One-way analysis is presented in box plots with individual subjects represented as circles (*clear circles* = control subjects, *red circles* = patients with Alzheimer’s disease [AD]). Plasma (**a**) and cerebrospinal fluid (CSF) (**b**) levels of KLK6 were compared between control subjects (*white box*) and patients with AD (*red box*). **c**, **d** Linear correlation analysis between KLK6 values (*y*-axis) and age (*x*-axis) for control subjects (*left*, *black correlation lines*) and patients with AD (*right*, *red correlation lines*). *NS* Not significant

We first examined whether KLK6 concentrations in plasma and CSF varied between control subjects and patients with AD in the cross-sectional cohort (Fig. 1a, b). In general, KLK6 concentrations in plasma were found to be approximately 100 times lower than in the CSF. We found a 30% increase in plasma levels of KLK6 (*p* = 0.0003) in the AD group compared with the control group, whereas CSF KLK6 levels did not differ significantly between the groups before or after applying age correction (mandated by the results presented below) (Fig. 1a, b).

### KLK6 correlation with age in cohort 1

Potential correlations between KLK6 and age were assessed in the control group as representing normal aging to examine whether KLK6 is an age-related protease. In control subjects, plasma levels showed no correlation with age, whereas CSF levels showed a trend of a positive association with age (Fig. 1c, d). In the AD group that included advanced AD cases (MMSE scores 4–18 = severe cognitive impairment), plasma but not CSF levels were linked to age (Fig. 1c, d).

### KLK6 associations with *APOE* ε4 status, MMSE scores and CSF AD biomarkers in cohort 1

**Fig. 2:**
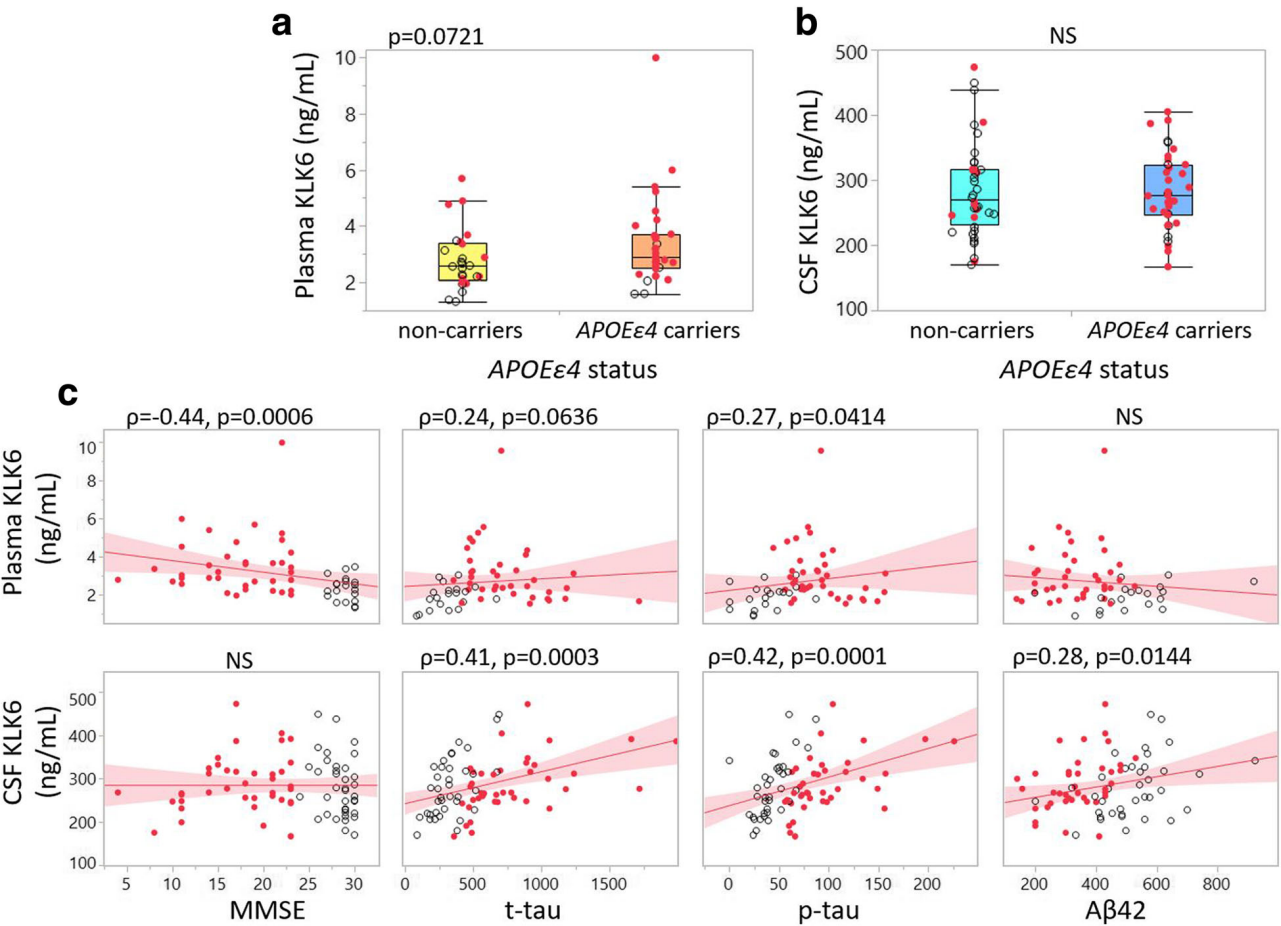
Association of kallikrein 6 (KLK6) to apolipoprotein E (*APOE*) ε4 allele status, clinical diagnosis and core Alzheimer’s disease (AD) biomarker profile in cohort 1. **a** Box plot of plasma KLK6 in non-carriers (*yellow box*) and *APOE* ε4 carriers (*orange box*). **b** Box plot of cerebrospinal fluid (CSF) KLK6 in non-carriers (*light blue box*) and *APOE* ε4 carriers (*dark blue box*). **c** Bivariate plots showing linear correlations between plasma (*upper panel*) and CSF (*lower panel*) with Mini Mental State Examination (MMSE) scores, total tau (t-tau), phosphorylated tau (p-tau) and amyloid-β 1–42 (Aβ_42_). *Clear dots* = control subjects, *red dots* = patients with AD. *NS* Not significant

We further assessed a potential association between KLK6 and *APOE* ε4 allele status. Plasma levels of KLK6 showed a trend towards an increase of 12% in *APOE* ε4 carriers compared with non-carriers (*p* = 0.0721), whereas no such tendency was found for CSF levels (Fig. 2a, b). On one hand, correlation analysis revealed that plasma levels of KLK6 were negatively correlated with MMSE scores and positively, though weakly, with p-tau, but not with other biomarkers. On the other hand, CSF KLK6 levels were not correlated with MMSE score but showed a positive correlation with t-tau, p-tau and Aβ_42_ (Fig. 2c).

**Fig. 3:**
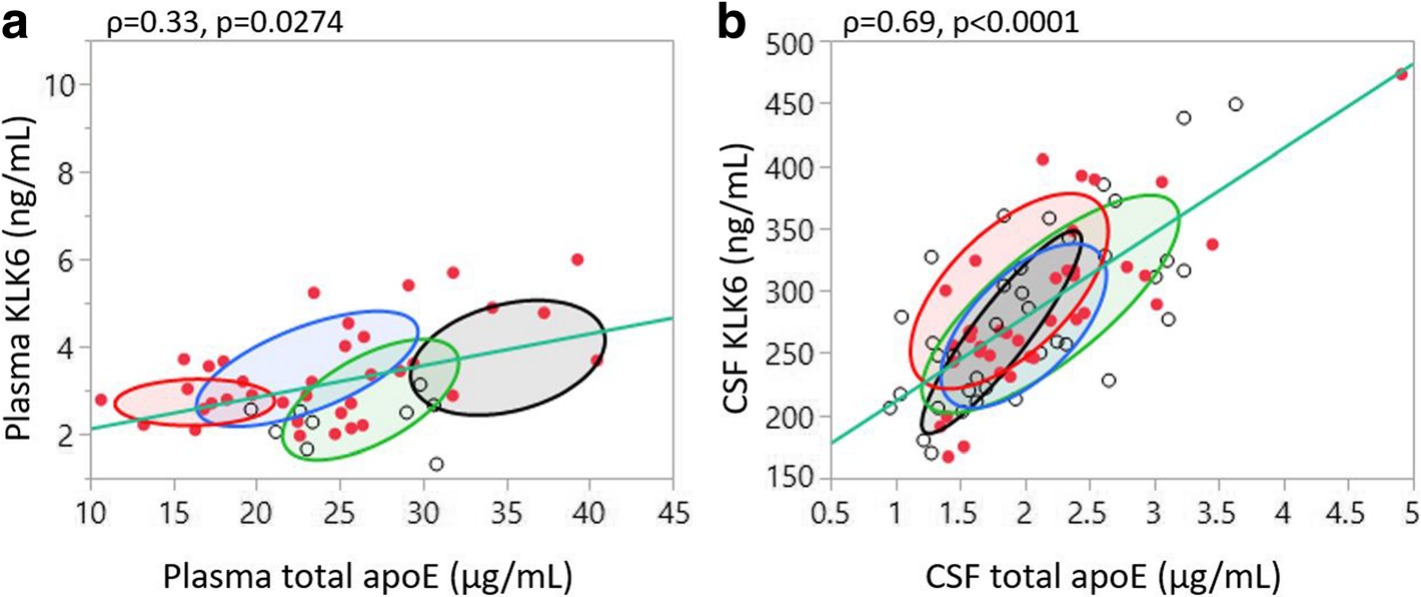
Kallikrein 6 (KLK6) association with total apolipoprotein E (apoE) levels assessed in cohort 1. **a**, **b** Scatterplots showing associations between plasma and cerebrospinal fluid (CSF) KLK6 with total apoE in respective fluids. Subjects were grouped according to their *APOE* genotypes, and normal ovals were drawn to represent the area covering the central 50% of the subjects in that group. *Red oval* = *APOE* ε4/ε4 genotype, blue oval = *APOE* ε3/ε4, *green oval* = *APOE* ε3/ε3, *black oval* = *APOE* ε2/ε3. *Clear dots* = control subjects, *red dots* = subjects with Alzheimer’s disease

Plasma and CSF total apoE levels were previously reported for cohort 1 [20]. In the present study we used those results to analyse potential correlations between KLK6 and total apoE levels in the respective body fluids, grouping the subjects on the basis of their *APOE* genotype. In both plasma and CSF, KLK6 correlated positively with total apoE levels (Fig. 3a, b). As previously reported [20], we noticed an association between *APOE* genotype and plasma apoE levels, such that *APOE* ε4 homozygous individuals (*red oval*) had the lowest levels and *APOE* ε4 non-carriers (*green* for *APOE* ε3/ε3 and *black* for *APOE* ε2/ε3) had the highest levels of total apoE. Plasma KLK6 levels remained similar between different *APOE* genotypes (Fig. 3a). There was no effect of *APOE* genotype on either CSF apoE or KLK6 levels (Fig. 3b). Interestingly, we did find a significant correlation between plasma KLK6 levels and plasma apoE levels only in the *APOE* ε3/ε4 carriers (Spearman’s rho = 0.450, *p* = 0.047), whereas CSF apoE and KLK6 levels were strongly correlated in all *APOE* genotype groups except for subjects with an *APOE* ε4/ε4 genotype (*APOE* ε2/ε3 Spearman’s rho = 0.893, *p* = 0.007; *APOE* ε3/ε3 Spearman’s rho = 0.706, *p* < 0.000; *APOE* ε3/ε4 Spearman’s rho = 0.697, *p* < 0.001).

### Lower CSF levels of KLK6 in patient groups in cohort 2

**Fig. 4:**
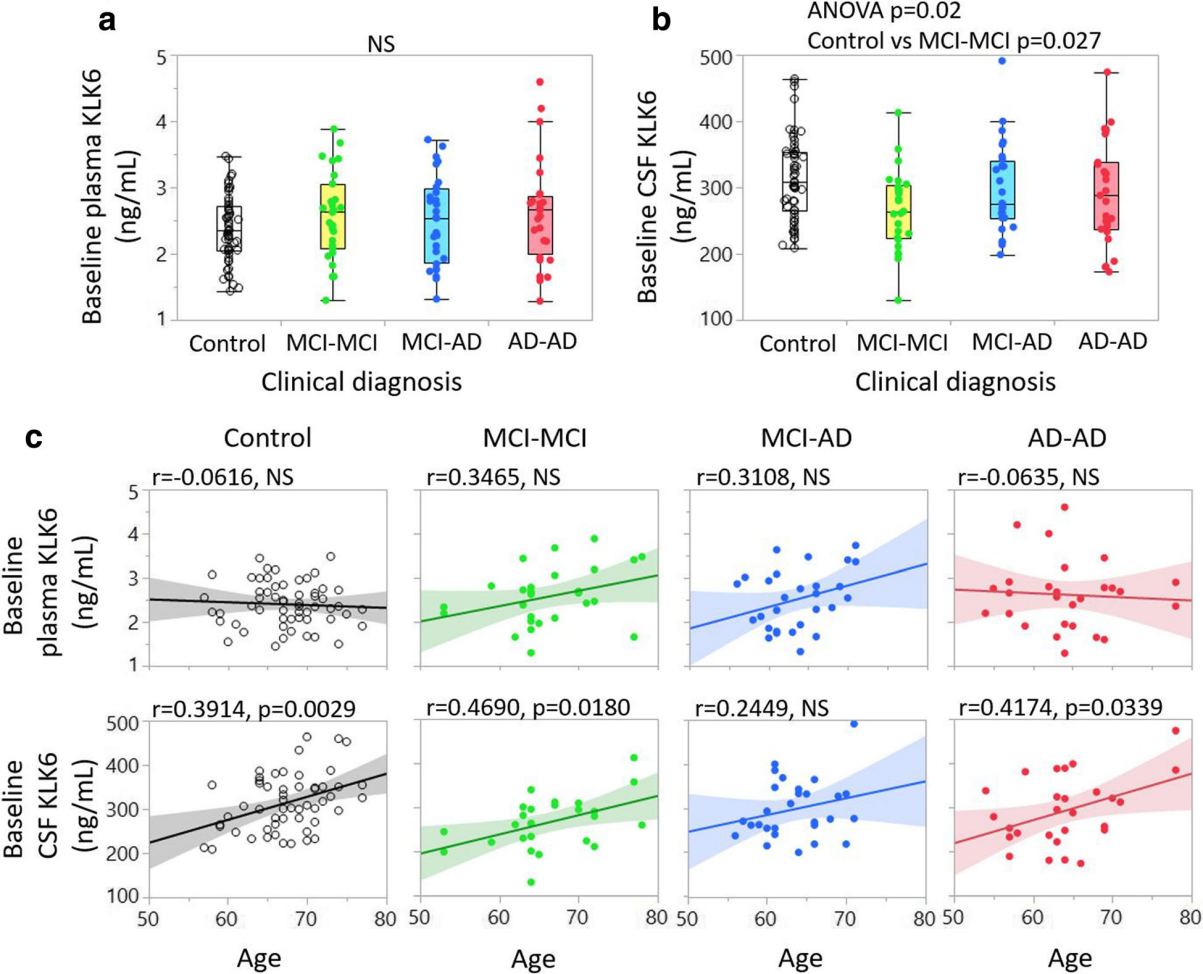
Kallikrein 6 (KLK6) levels among subject groups and association with age in cohort 2. One-way analysis is presented in box plots with individual subjects represented as dots. Plasma (**a**) and cerebrospinal fluid (CSF) (**b**) levels of KLK6 are compared between control subjects (*white boxes*), patients with mild cognitive impairment that remained unchanged over 24 months (MCI-MCI) (*yellow boxes*), patients with mild cognitive impairment who developed Alzheimer’s disease (MCI-AD) (*blue boxes*) and patients with Alzheimer’s disease (AD) (*red boxes*). **c**, **d** Linear correlation analysis between baseline plasma KLK6 (**c**) and baseline CSF KLK6 (**d**) values (*y*-axis) and age (*x*-axis). *Black correlation lines* = control subjects; *green dots*, *green correlation lines* = patients with MCI-MCI or patients with MCI stable over 24 months; *blue dots*, *blue correlation lines* = MCI-AD (MCI converters to AD = *Clear dots*); *red dots*, *red correlation lines* = AD from baseline. *NS* Not significant, *ANOVA* Analysis of variance

The subjects included in cohort 2 were grouped according to the final clinical diagnosis at the 24-month follow-up (MCI-MCI, MCI-AD and AD-AD) as described previously [23]. At baseline, patients (patients with MCI and patients with AD together) showed a 10% decrease in CSF levels of KLK6 versus control subjects (*p* = 0.01) (not shown). Upon separation of the subjects into the four groups, CSF KLK6 levels differed significantly between the groups as assessed by ANOVA (*p* = 0.02). This significant difference remained when we performed pairwise comparisons between the diagnostic groups while correcting for the age difference by use of ANCOVA. The analysis revealed a significant decrease in the CSF KLK6 levels of the MCI-MCI group versus control subjects (*p* = 0.027 after Bonferroni correction for six comparisons). Plasma levels in cohort 2 did not differ significantly between the groups even after correcting for age (Fig. 4a, b).

### KLK6 correlations with age in cohort 2

Baseline plasma and CSF KLK6 levels were analysed for correlations with age in the individual diagnostic groups. We observed no significant correlation between plasma KLK6 and age in any subject group; however, consistent with our findings in cohort 1, CSF KLK6 levels showed a significant positive correlation with increasing age in control subjects but also in the MCI-MCI and AD-AD groups (Fig. 4c, d).

### KLK6 correlations with *APOE* status, MMSE score and AD biomarkers in cohort 2

**Fig. 5:**
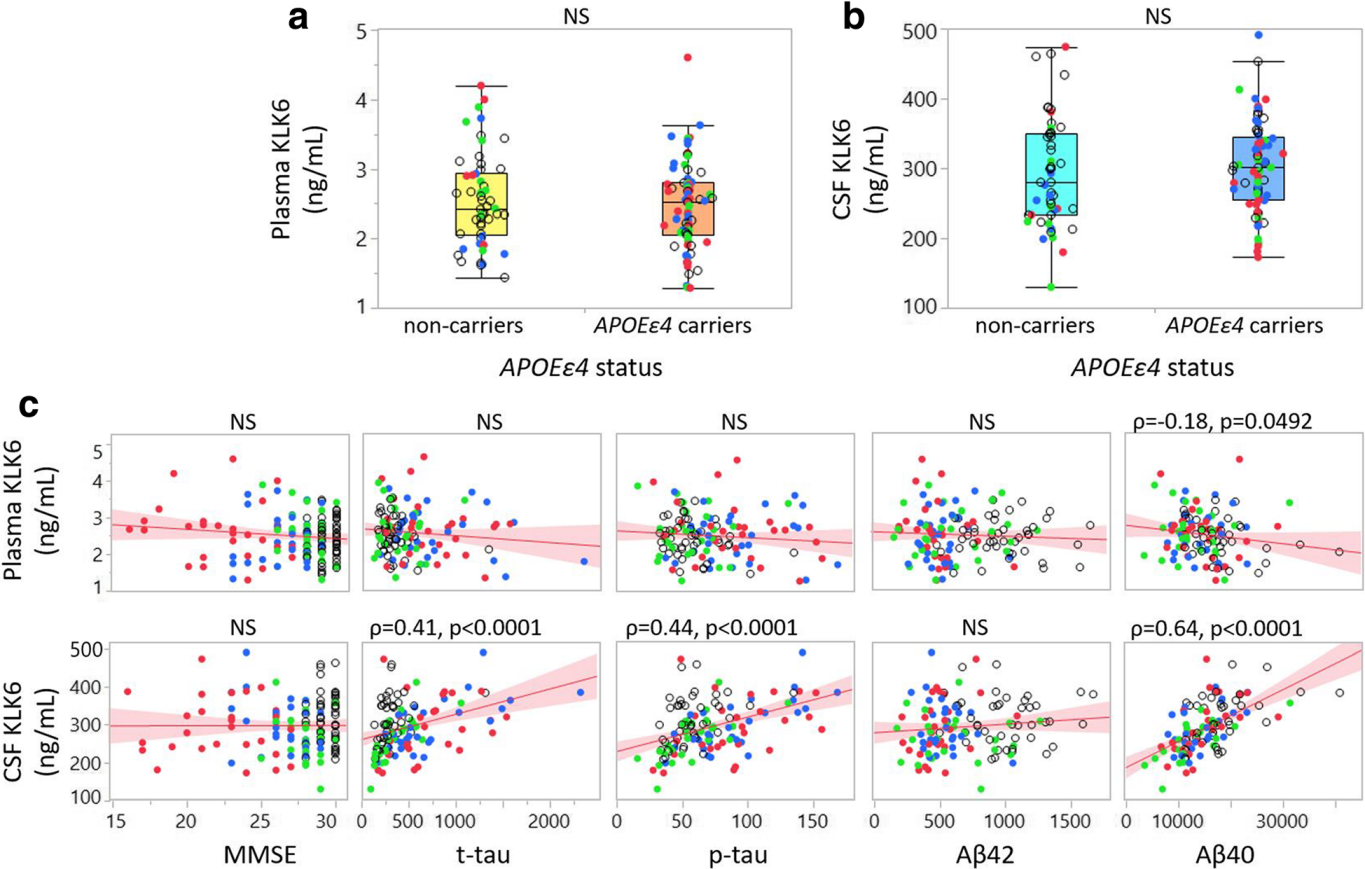
Association of kallikrein 6 (KLK6) to apolipoprotein E (*APOE*) ε4 status, clinical diagnosis and core Alzheimer’s disease (AD) biomarker profile in cohort 2. **a** Box plot of plasma KLK6 in non-carriers (*yellow box*) and *APOE* ε4 carriers (*orange box*). **b** Box plot of cerebrospinal fluid (CSF) KLK6 in non-carriers (*light blue box*) and *APOE* ε4 carriers (*dark blue box*). **c** Bivariate plots showing correlations between plasma (*upper panels*) and CSF (*lower panels*) with Mini Mental State Examination (MMSE) scores, total tau (t-tau), phosphorylated tau (p-tau), amyloid-β 1–42 (Aβ_42_) and amyloid-β 1–40 (Aβ_40_). *Open dots* = control subjects; *green dots* = patients with mild cognitive impairment that remained unchanged over 24 months; *blue dots* = patients with mild cognitive impairment who developed Alzheimer’s disease; *red dots* = AD. *NS* Not significant

In cohort 2, associations could not be found between KLK6 fluid levels and *APOE* ε4 status (Fig. 5a, b). Because apoE levels had not been quantified for this cohort, we were unable to assess potential relationships between total apoE and KLK6 levels. No significant correlation was observed between KLK6 levels and MMSE scores. Plasma levels of KLK6 did not correlate with t-tau, p-tau or Aβ_42_, though a weak negative correlation was found between plasma KLK6 and Aβ_40_ (Fig. 5c). CSF KLK6 levels, however, showed strong positive correlations with t-tau, p-tau and Aβ_40_, but not with Aβ_42_ (Fig. 5c).

### Longitudinal assessment of KLK6 levels in cohort 2

**Fig. 6:**
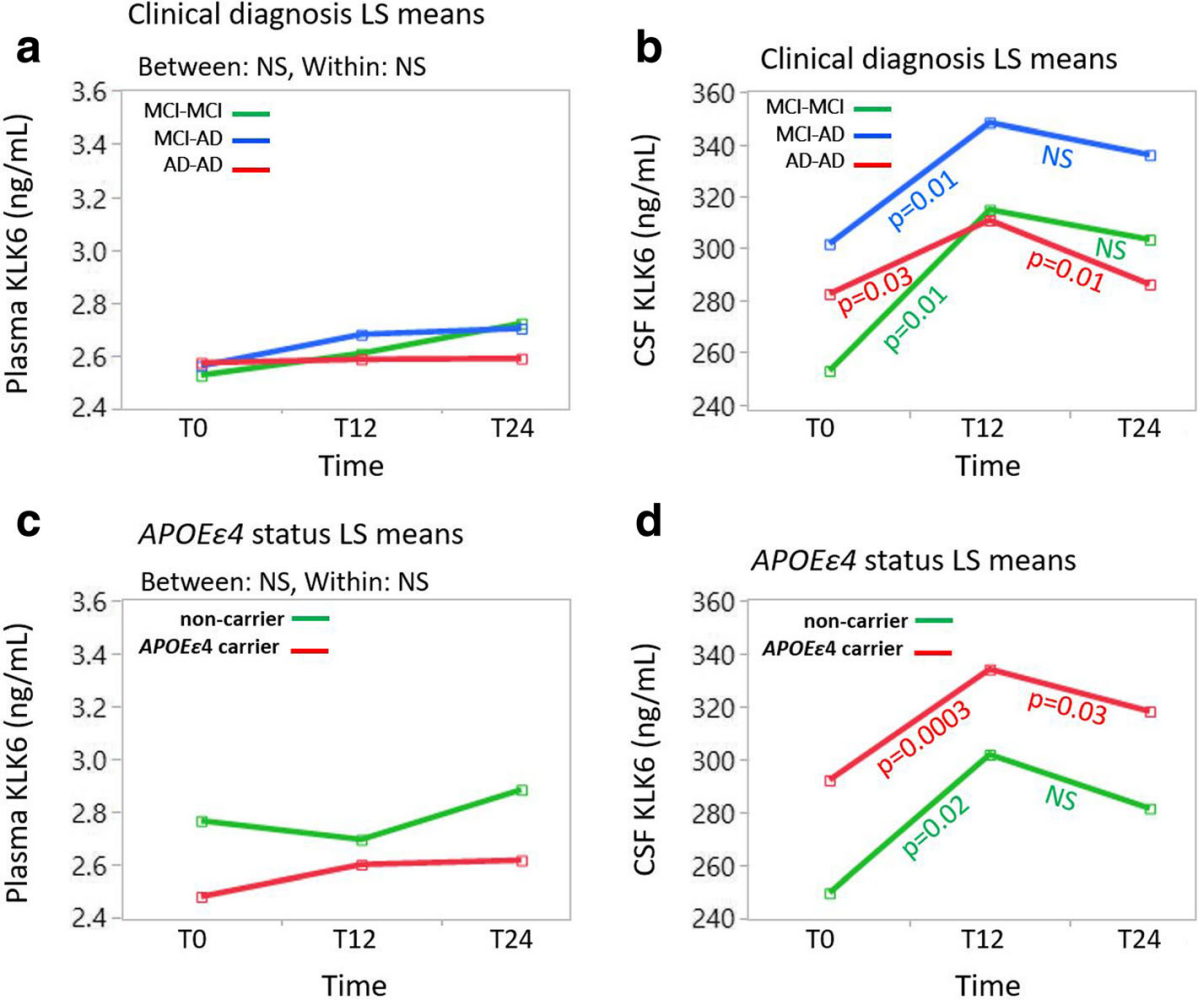
Longitudinal analysis of kallikrein 6 (KLK6) among patient groups as well as among apolipoprotein E (*APOE*) ε4 carriers and non-carriers. **a**, **b** Multivariate analysis of variance (MANOVA) plots show vector graphs taking least squares (LS) mean values from each category (*green lines* = patients with mild cognitive impairment that remained unchanged over 24 months [MCI-MCI]; *blue lines* = patients with mild cognitive impairment who developed Alzheimer’s disease [MCI-AD]; *red lines* = patients diagnosed with Alzheimer’s disease at inclusion [AD-AD]) in the *y*-axis over three time points (T0, T12 and T24, representing baseline and after 12 and 24 months, respectively). **c**, **d** MANOVA plots between subjects grouped as *APOE* ε4 carriers (*red lines*) and non-carriers (*green lines*). Significant alterations are reported with *p* values after Bonferroni correction (*n* = 3 comparisons) derived from pairwise comparisons between T0 and T12 and between T12 and T24 by Student’s *t* test. *NS* Not significant

Longitudinal assessment of KLK6 levels was performed using MANOVA comparing the least squares means of all data points among patient groups at baseline (T0) and at 12-month (T12) and 24-month (T24) follow-up. No significant alteration in plasma levels of KLK6 from baseline to 12 or 24 months was found in any of the groups (Fig. 6a). The CSF levels did not differ among subjects MCI-MCI, MCI-AD or AD-AD at any time point. However, patients with MCI-MCI and patients with MCI-AD exhibited a significant increase in CSF KLK6 levels from T0 to T12 (MCI-MCI, *p* = 0.0111; MCI-AD, *p* = 0.0092) and maintained higher levels at T24. On the contrary, patients with AD-AD showed an increase in CSF KLK6 levels from T0 to T12 (*p* = 0.0269), followed by a decrease from T12 to T24 (*p* = 0.0126) (Fig. 6b). When the patients were grouped according to their *APOE* ε4 status, again, plasma levels remained similar between the two groups as well as between the time points within each group (Fig. 6c). CSF levels were significantly increased at T12 compared with T0 (non-carriers, *p* = 0.0175; *APOEε4* carriers, *p* = 0.0003) but decreased from T12 to T24, though this decrease in CSF levels was significant only for the *APOE* ε4 carriers (*p* = 0.0327). (Fig. 6d).

## Discussion

In the present study we aimed to analyse the potential of the age-related protease KLK6 as a biomarker for AD. With a comprehensive study design we have used the same methodology to investigate two separate cohorts—a cross-sectional and a longitudinal study—for KLK6 levels in both plasma and CSF. As expected, we found that the levels of CSF KLK6 were positively related to age in control individuals, validating previous data suggesting CSF levels of KLK6 as a marker for healthy aging [14]. The results derived from our analyses endorse two previous reports, one by Menendez-Gonzalez [18] and one by Wennström [19] and colleagues, showing that CSF levels of KLK6 are not useful as a biomarker for AD. We further show that CSF KLK6 levels were not useful for identifying patients with MCI transitioning into an AD diagnosis over the 24-month period investigated. Importantly, our study results propose increased levels of plasma KLK6 in patients with AD with a more advanced disease stage (cohort 1). Increased plasma levels of KLK6 in these individuals also correlated with lower MMSE scores, supporting the notion that an increase in plasma KLK6 levels might be associated with cognitive decline. This statement was further supported by the lack of an age-associated increase in plasma KLK6 in the control group, corroborating our reasoning that increased plasma levels of KLK6 may indicate a pathological aging process.

Importantly, under normal aging there was no correlation between plasma KLK6 levels and age in either cohort. In fact, the regression slopes between age and the plasma KLK6 data in control subjects versus patient groups were different. Hence, despite the significant age difference between the control subjects and patients with AD in group 1, we were unable to adjust the outcome of our analysis for age. Instead, enabled by the similar regression slopes between age and plasma KLK6 in the AD patient groups in both cohorts, which differed significantly in age (median age of 78 years in cohort 1 versus 64 years in cohort 2; *p* < 0.0001), we found that an age-corrected comparison of plasma KLK6 levels in the AD groups of the two cohorts yielded significantly higher plasma levels in the patients with AD included in cohort 1. The latter AD group differed significantly from the AD group of cohort 2 in regard to disease severity (*p* < 0.0001). These results support an assumption that plasma KLK6 levels are associated with AD disease severity.

The identification of significant correlations between CSF levels of KLK6 and levels of the conventional CSF AD biomarkers in both cohorts, although in different directions for Aβ_42_, underlines a potential involvement of KLK6 in the pathological cascade of events leading to AD. Levels of KLK6 in the CSF from subjects in both cohorts showed a strong and significant correlation with CSF levels of t-tau and p-tau. The conflicting results of either positive or negative associations between CSF KLK6 levels and Aβ_42_ levels between cohort 1 and cohort 2 further suggest a potential involvement of KLK6 in AD pathological processes at different stages of disease progression, with patients with AD in cohort 1 being both older and more advanced in their cognitive dysfunction than the patients with AD in cohort 2. Whether the levels of KLK6 per se, the composition of total KLK6 and the pool of active KLK6 are of importance for AD-related pathways needs to be determined in future studies.

KLK6 is generated as a pre-pro-enzyme that becomes active through cleavage of the pre- and pro-peptides by matrix metalloproteinases and proteases of the thrombostasis axis, both of which co-exist with KLK6 in brain tissue [28, 29]. In CSF, KLK6 was earlier found to be mostly of the pro-form [30], but the specific activity of this form needs to be elucidated. Our analyses in the present study provided only the total KLK6 levels in plasma and CSF without regard to activity, specific pro-forms or KLK6 isoforms. Hence, future studies need to address whether the balance between the inactive and active forms of KLK6 is altered during the development of AD. Three different KLK6 isoforms have been described [31], generated as a result of alternative splicing. The classic KLK6 comprises the full-length pre-pro-enzyme and is predominant in nervous tissue, whereas the other two isoforms are truncated proteins. It is noteworthy that the alternate isoforms may constitute 10–20% of total KLK6. With our detection method, we used monoclonal antibodies generated against full-length recombinant human KLK6 protein, so we cannot rule out the simultaneous detection of alternative isoforms. Whether the truncated isoforms are functional proteins, and if so, whether they have activity similar to that of the full-length KLK6, are yet to be investigated.

We cannot properly explain the involvement of *APOE* ε4 in our results. However, a recent study by Tamboli and colleagues showed that a secreted serine protease (other than thrombin and cathepsin G) can proteolytically yield fragmented apoE and that the activity of this protease can be inhibited by the astrocyte-secreted serine protease inhibitor α_1_-antichymotrypsin (ACT) [32]. The exact identification of this protease remains to be determined, although speculating that this protease may be KLK6 is not too far-fetched. It was previously found that approximately 5% of the active KLK6 in human milk and ascites fluid was stably bound to ACT, whereas KLK6 in serum and CSF was found to be free and in an uncomplexed form [30]. More recently, however, it was found that the serine protease α_1_-antitrypsin (AAT) is the main inhibitor of KLK6 in biological fluids [33]. Both of these described findings are of relevance when considering the previously reported increase in plasma KLK6 and CSF levels of both ACT and AAT in patients with AD [34]. Importantly, the same study further showed that patients with dementia with Lewy bodies (DLB) also exhibited increased levels of CSF but not plasma ACT and AAT. That patients with DLB in a later study were found to have significantly lower CSF KLK6 levels than both patients with AD and control subjects [19] unfortunately complicates a conclusion. Importantly, the relevance of the activity of the different pools of KLK6 in the periphery versus the central nervous system is unknown, and it may be of importance if speculating that KLK6 is an apoE-cleaving enzyme. We found in both cohorts that the two pools of KLK6 were not correlated (data not shown), which could have resulted from the fact that plasma KLK6 and CSF KLK6 have different sources of production [11]. To our knowledge, we are the first to report a correlation between *APOE* ε4 and KLK6 levels, which was particularly evident in the CSF. The biological significance of these findings is currently being addressed in our laboratory.

Last, with the opportunity provided through the longitudinal nature of cohort 2, we were able to assess whether plasma and CSF KLK6 levels increase over time (24 months) in (1) patients with MCI that was stable over 2 years or in those converting to AD and (2) patients with AD. Although CSF levels of KLK6 were altered between time points, they followed a similar trend in the investigated patient groups and were not related to *APOE* ε4 status or cognition. Hence, alterations in CSF KLK6 levels within a 24-month follow-up period could not differentiate patients with AD from amnestic MCI and MCI converters in the age group 60–65 years.

## Conclusions

Plasma KLK6 levels may be of value as a biomarker only for patients with advanced AD. On the basis of identified correlations between KLK6 and CSF AD biomarkers, we further conclude that although CSF KLK6 levels are not useful as a biomarker for AD per se, they are related to AD neuropathological processes, and that this association may change over time as disease progresses. At last, we have identified an association suggesting some convergence between apoE and KLK6 that may be based on *APOE* genotype. Future studies need to assess KLK6 activity and its relevance both to AD and to the AD risk allele *APOE* ε4.

## Abbreviations

AAT: α_1_-Antitrypsin
ACT: α_1_-Antichymotrypsin
AD: Alzheimer’s disease
AD-AD: Patients diagnosed with Alzheimer’s disease at inclusion
ANCOVA: Analysis of covariance
ANOVA: Analysis of variance
*APOE*/apoE: Apolipoprotein E
Aβ_40_: Amyloid-β 1–40
Aβ_42_: Amyloid-β 1–42
CSF: Cerebrospinal fluid
DLB: Dementia with Lewy bodies
ELISA: Enzyme-linked immunosorbent assay
KLK6: Kallikrein 6
LS: Least squares
MANOVA: Multivariate analysis of variance
MCI: Mild cognitive impairment
MCI-AD: Patients with mild cognitive impairment who developed Alzheimer’s disease
MCI-MCI: Patients with mild cognitive impairment that remained unchanged over 24 months
MMSE: Mini Mental State Examination
NINCDS-ADRDA: National Institute of Neurological and Communicable Disorders and Stroke-Alzheimer’s Disease and Related Disorders Association
p-tau: Phosphorylated tau
t-tau: Total tau
